# Dental Caries during Pregnancy

**Published:** 1896-06

**Authors:** H. F. Vandervoort


### Dental Caries during Pregnancy.

BY H. F. VANDERVOORT, D.D.S.

Read before the Odontological Society of Cincinnati.

It is generally accepted that the teeth of women are more liable to become carious, during pregnancy, and while this condition has been much talked about I do not find anything recorded on the subject showing the relationship between dental caries and pregnancy. I have made no record myself of such cases, but have taken this subject for my paper that I may learn something of a condition or subject that should have our attention. As for dental caries, I believe the chemico-parasitic theory, of which Dr. Miller is the exponent, is generally upheld, at any rate he has shown positively that under certain conditions there is a ferment of a vital or living nature present in the human mouth, and this ferment has the power of self-reproduction. The product of this ferment is lactic acid, which deprives the teeth of their lime salts, and these micro-organisms can live and flourish in the interior of the decalcified mass of tooth structure. Now we all know how they begin business. They first, of course, find a suitable location, which is, in some fissure, or at some point, not properly cleansed, where they secrete the acid, when the enamel is alternately decalcified, and the dentine reached which is soft, and then assisted by the dental tubuli, the interior of the tooth is very quickly reached. This is a brief description of the theory and shows that the influences are directed from the exterior, but in the case of pregnant females, I think the influences are directed from the exterior and interior both, and at the same time, it is said it is due to the neglect or a lack of attention at that time. That certainly has a great deal to do with it, but we find that the patients suffer from ocular trouble and numerous other nervous disorders, and while the condition generally excites a high nervous tension, or we might say, is very displeasing to the nervous system when the patient is easily excited and worried and is troubled with nausea. Now we find a change in the oral secretions which furnishes a more suitable soil for the development of micro-organisms, and from lack of attention such as proper cleansing and use of suitable mouth-wash the organisms have nothing to interfere with their progress of destruction, and from the nervous condition nature is weakened and is unable to protect the organs from within, it is thought by some that through the demands of the developing fetus the lime salts are abstracted from the teeth ; this, however, I do not believe, for my knowledge of the histology of teeth does not show any place of absorbing the lime salts from them or how carried from the teeth to the fetus, it may be done, yet since the teeth receive nutrition from the blood it would seem that nature was cruel indeed to cease giving nutrition and even rob them of so important a part. It is also thought that if the teeth were deprived of nutrition other bones would suffer too, has it been proven they do not? I think, probably, they do, but on account of the predisposition of the teeth to caries they suffer most. Now, to conclude, I will say the family physician generally knows of the condition first and I have avoided mention of treatment, for generally when we are called on, we only have to treat acute conditions and the treatment would be governed by the case in hand.

